# Safety and Mode of Action of Diabetes Medications in comparison with 5-Aminolevulinic Acid (5-ALA)

**DOI:** 10.1155/2019/4267357

**Published:** 2019-11-06

**Authors:** Peter R. Rehani, Hanaa Iftikhar, Motowo Nakajima, Tohru Tanaka, Zaid Jabbar, Riyadh N. Rehani

**Affiliations:** ^1^University of Wisconsin-Madison, Madison WI, USA; ^2^Royal College of Surgeons in Ireland, Bahrain; ^3^SBI Pharmaceuticals Co. Ltd., Tokyo, Japan; ^4^DuPage Medical Group, Chicago, IL, USA; ^5^SBI ALApharma Co. Ltd., Hong Kong

## Abstract

5-Aminolevulinic acid (5-ALA) is a delta amino acid naturally present in every living cell of the human body. 5-ALA is produced in the mitochondria as the first product of the porphyrin synthesis pathway and composes heme; exogenously supplemented 5-ALA helps in upregulating mitochondrial functions. Mitochondrial dysfunction has been associated with the pathophysiology of diabetes mellitus. Thus, in this review, we evaluate the mechanisms of action and adverse effects of common medications used to treat type 2 diabetes mellitus as well as 5-ALA including its mechanism and possible use in diabetes management.

## 1. Introduction

Diabetes mellitus is a multidimensional disease with various contributing factors including genetics, diet, race, and lifestyle; prevalence has steadily increased, peaking in 2014 with 108 million cases worldwide. Type 1 diabetes mellitus causes insulin dependence due to the pancreas producing very little to no insulin, thereby requiring an exogenous supply of insulin. Type 2 diabetes mellitus often develops due to obesity and lack of physical activity, which results in insulin resistance and can lead to decreased production of endogenous insulin as the disease progresses. Insulin is needed to regulate blood glucose levels, and diabetes mellitus results from the lack of glucose regulation. The abnormally high glucose levels that persist eventually cause long-term irreversible damage in the human body. The effects of persistent high blood glucose levels have been well documented and include atherosclerosis and neuropathy. This culminates in a wide variety of end organ damage ranging from coronary artery disease, skin infections, and strokes, to microvascular complications including diabetic nephropathy, peripheral neuropathies, and retinopathies [1, 2]. Diabetes mellitus can present with vague symptoms such as tiredness, thirst, hunger, headaches, blurred vision, increased urination, weight loss, pain or tingling sensations, and skin infections. The diagnosis is made with random or fasting blood glucose levels, as well as glycated hemoglobin (HbA1c) levels. Following diagnosis, patients are counselled on the importance of diet, exercise, and weight loss (if applicable), and eventually most are started on medication. First-line therapy for patients with type 1 diabetes is insulin therapy, whereas the medication options available for patients with type 2 diabetes are far broader, up to and including insulin therapy. Diabetes is diagnosed based on an HbA1c of greater than 6.5%, a fasting blood glucose reading more than 6.9 mmol/L, or a random blood glucose reading more than 11 mmol/L. The HbA1c levels are generally used as a guide for the glycemic control in treating diabetic patients, with levels less than 7% as the common target and a more flexible target of less than 8% for older patients or those with comorbidities. Type 1 diabetes patients are asked to monitor their home glucose readings as well, including their fasting and random blood glucose readings, which determine the need for insulin dose adjustments [3–6].

### 1.1. Diabetes Management-Targeting Pathways

**Figure 1:**
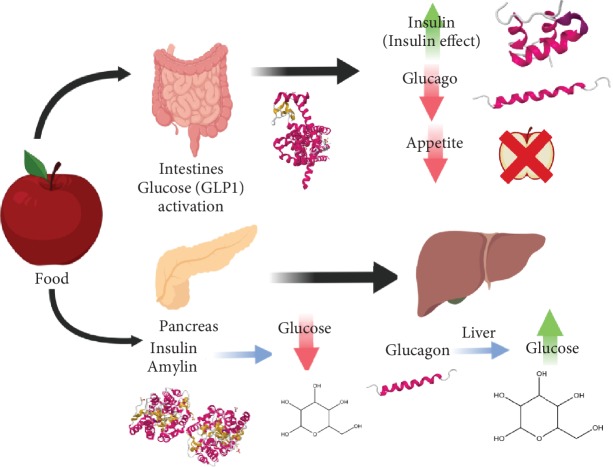
After food is consumed and digestion begins, glucose levels start to increase, as do other hormones such as glucagon-like peptide (GLP-1) which is released in the intestines. Glucagon-like peptide 1 (GLP-1) is an incretin, which works by triggering insulin production (as insulin acts to decrease glucose levels) and inhibiting glucagon production (glucagon acts to increase glucose levels). This occurs to counteract the increased glucose, and it induces the feeling of satiety and reduces apatite by sending signals to the brain that one is full [44]. The consumption of food also triggers the release of pancreatic hormones like insulin, amylin, and glucagon. Insulin and amylin both work to decrease glucose levels and inhibit glucagon while glucagon acts on the liver to raise glucose levels.

The targeted therapies for patients with type 2 diabetes are established based on various pathways through which glucose control can be achieved (see Figure 1).

## 2. Diabetes Medications

The American Diabetes Association's Standards of Medical Care in Diabetes Patients [7] specifies the pharmacological management of type 2 diabetes, all of which are discussed below.

### 2.1. Metformin (Type: Biguanide)

Metformin is the most common initial drug prescribed for type 2 diabetes in the world [8]. Metformin acts via an antihyperglycemic pathway through an increased glucose tolerance in patients with type 2 diabetes. Typically, this is measured via blood plasma glucose levels and postprandial plasma levels. As an antihyperglycemic agent, the mode of action entails decreasing hepatic glucose production and the intestinal absorption of glucose and increasing peripheral glucose uptake and utilization. It also helps break down free fatty acids by activating adenosine monophosphate- (AMP-) activated protein kinase in hepatocytes. Additionally, metformin does not affect insulin secretion levels; fasting insulin levels and 24-hour plasma response have been shown to actually decrease. Metformin is secreted without additional metabolization through tubular secretion in the kidney, and because of this, renal issues can prove fatal through lactic acidosis—a rare metabolic complication. This is caused by the inhibition of hepatic gluconeogenesis by inhibiting a mitochondrial isoform of glycerophosphate dehydrogenase (mGPD), preventing glycerol from participating in the gluconeogenic pathway. Finally, nicotinamide adenine dinucleotide (NADH) accumulates, and the conversion of lactate to pyruvate slows. Care must be taken with older populations, specifically with renal function monitoring. Metformin differs from other medications by not producing hypoglycemia or hyperinsulinemia in type 2 diabetic patients. Lastly, GI disturbances and decreases in appetite (and subsequent weight loss) are some side effects [9–11].

### 2.2. Sulfonylurea (Glipizide, Gliclazide, and Glimepiride)

Sulfonylureas are only beneficial to patients who have retained some degree of residual pancreatic beta cell functionality, as they work by stimulating insulin secretion. This is often difficult due to the death/loss of function of the insulin-producing beta cells in the pancreas which accompanies diabetes. The necessity for beta cells' presence stems from the molecular mechanism of action: on the surface of these cells, sulfonylurea has specific neuronal receptors. When a sulfonylurea-type molecule binds, it causes the cellular membrane to depolarize, leading to the calcium channel opening and resultant calcium influx. Next, this change in the charge of the cell causes actomyosin filaments to contract and, in turn, release insulin from the cell. After a brief period, insulin granule transmission begins, and new insulin granules are formed. Sulfonylureas' target receptor is a complex of the sulfonylurea 1 receptor (SUR1), specifically the K-ATP channel, altering the resting potential. Adding 5-ALA has been shown to aid this process, especially with improved results in patients with consistent insulin resistance by increasing the availability of ATP, which aids with key metabolic processes (like the TCA cycle) within the mitochondria [10]. Sulfonylureas are glucose-level independent, meaning that there is a higher sensitivity to amino acids and, in turn, higher insulin release. An increased sensitivity of beta cells to glucose and nonglucose secretagogues develops; thus, hypoglycemia and weight gain are resultant potential side effects. Finally, an increase in peripheral glucose utilization has been noted with this drug class by both stimulating hepatic gluconeogenesis and increasing the number/sensitivity of insulin receptors [12–15].

### 2.3. Meglitinides (Repaglinide and Nateglinide)

Meglitinides act on different beta cell receptors, but in a similar fashion to sulfonylureas. They work on the same K-ATP channels and increase insulin secretion. One potential downside is the lower binding affinity present at the surface level of the pancreatic beta cells; combined with the faster dissociation rates, the efficacy of this class of drugs is less than its parallel. Their adverse effects include hypoglycemia and weight gain. Lastly, they are more expensive than sulfonylureas and are commonly used in patients with allergies to the former [16–18].

### 2.4. Thiazolidinediones (Rosiglitazone and Pioglitazone)

Thiazolidinediones (TZDs) effectively attempt to mimic insulin by reducing hyperglycemia even with an impaired insulin tolerance. This leads to substantial reductions in hyperinsulinemia, which is caused by an increase of peripheral glucose consumption and decrease in hepatic glucose levels. There is no change in the secretion levels on insulin, but potential restoration of pancreatic beta cell insulin reserves has been observed. The exact mode of action has not been specified; however, there are two key effects to be discussed. First, the affinity of TZDs to the binding site known as peroxisome proliferator-activated receptor- (PPAR-) gamma on the adipocyte protein 2 (aP2) molecule, a key gene involved in weight loss efforts, has led to the connection between the TZD hypoglycemic action and the promoter region PPAR-gamma, especially with the PPAR-gamma agonist rosiglitazone. Ultimately, insulin sensitivity is not a direct by-product of this aspect, which raises the second point—TZDs are able to uniquely activate the phosphoinositide 3-kinase (PI3K) pathway with or without PPAR-gamma. Clinically, rosiglitazone and pioglitazone have distinct levels of PPAR-gamma activation (as agonists but the latter with a considerably lower effect). In both cases, however, it has been shown that activation of PI3K phosphorylation of AKT occurs, leading to the increased insulin sensitivity. Additional work has suggested that PPAR-gamma is still necessary for survival due to the offsite activity. This duality may be caused by a separate mitochondrial binding site that TZDs bind to, leading to the insulin sensitization. One side effect observed in clinical trials is increased food intake, which may lead to weight gain (with rosiglitazone). TZDs also have an increased risk of hypoglycemia and bone fractures. Furthermore, they cause edema and are contraindicated in patients with New York Heart Association class III and IV heart failure. Rosiglitazone was found to increase the risk of myocardial infarctions and was banned in Europe in 2010. Pioglitazone, on the other hand, was shown to cause an increased risk of bladder cancer, which led to the drug being banned in some countries. Although TZDs are approved for monotherapy, guidelines advise them to be considered as add-on combination therapy (e.g., with metformin or sulfonylureas) due to their associated risks and taking into account their advantages/disadvantages [18–26].

### 2.5. GLP Agonists (Exenatide, Lixisenatide, Liraglutide, Albiglutide, and Dulaglutide)

Glucagon-like peptide (GLP-1), an incretin, is a gastrointestinal peptide involved in the regulation of glucose levels where the hormone is released upon consuming food. It stimulates insulin formation and release, and this occurs upon oral ingestion of food exclusively. GLP binds to receptors present in many tissues including beta cells, gastric mucosa, the kidney, the heart, etc. This hormone is targeted in diabetes because it causes insulin release from the beta cells, as well as slows down gastric emptying and inhibits excess glucagon release after meals. This, in turn, decreases appetite (causing weight loss). GLP agonists, however, are injectable medications that act by enhancing these effects in the body, thereby making it less appealing to some patients. This drug class is contraindicated in renal failure, multiple endocrine neoplasia type 2, or in those with a personal or family history of medullary thyroid cancer. Their potential (but rare) side effect includes pancreatitis, and they are also comparatively costlier versus other first-line medications. However, liraglutide, in specific instances, has been shown to reduce adverse cardiovascular events [25, 27, 28].

### 2.6. DPP4 Inhibitors (Sitagliptin, Saxagliptin, Linagliptin, and Alogliptin)

DPP4 is an enzyme that deactivates the glucose-dependent insulinotropic polypeptide (GIP) and GLP-1. The inhibition of this enzyme causes an increased availability of GLP-1 levels in the body (as seen above). DPP4 inhibitors are a group of drugs that are an oral GLP-1-based therapy; however, they are not as effective at glucose or weight reduction. Their potential side effects are angioedema and pancreatitis, but they have a lower risk of hypoglycemia. This class may be considered in those who are intolerant of or have contraindications to metformin, sulfonylureas, or thiazolidinediones, such as patients with chronic kidney disease or who are at a high risk of hypoglycemia. They can also be considered an add-on medication; however, this is often cost prohibitive [21, 25, 29].

### 2.7. Sodium Glucose Co-Transporter-2 Inhibitors (Gliflozins)

Sodium glucose co-transporter-2 (SGLT2) works by inhibiting the SGLT2 receptors in the kidneys' proximal convoluted tubule, the site where most glucose is reabsorbed back into the body. These medications prevent the reabsorption of glucose and increase its urinary excretion and can cause polyuria which, in certain cases, can result in postural hypotension. They also contribute to weight loss and some side effects that include urinary and genital infections, as well as diabetic ketoacidosis [21].

### 2.8. Alpha-Glucosidase Inhibitors (Acarbose, Miglitol, and Voglibose)

Alpha-glucosidase inhibitors are not included in the ADA recommendations, possibly due to their adverse effects—bloating, flatulence, and diarrhea—as well as the fact that they are less effective and have higher price points compared to other medications. They are oral medications that work by inhibiting the upper gastrointestinal enzymes (alpha-glucosidases), which convert polysaccharides into monosaccharides, and slow the intestinal absorption of glucose. In older patients with type 2 diabetes, acarbose may also increase insulin sensitivity; its contraindications include inflammatory bowel disease and liver cirrhosis [21, 25, 30].

### 2.9. Orlistat (Lipase Inhibitor)

Orlistat is a minimally absorbed drug that inhibits pancreatic and gastric lipases and results in blocking the absorption of about 30% of ingested fat, which can aid in improving glucose control in patients with diabetes—again, it is not considered a first-line therapy. Its adverse effects include abdominal pain, steatorrhea, and fecal urgency/incontinence and have also been implicated in kidney damage. Ultimately, it was designed for weight loss rather than diabetes, and as such, it may be used as an adjunct therapy to other diabetic drugs rather than as a first-line treatment [31, 32].

## 3. Overview: Pharmacological Management of Type 2 Diabetes

If, after 3 months, inadequate control persists (e.g., HbA1c not reaching target levels), an additional medication should be added after assessing for atherosclerotic cardiovascular disease (ASCVD). If the patient does have ASCVD, the ADA recommends using a drug with cardiovascular risk reduction (e.g., liraglutide and empagliflozin), taking into account each of their specific effects and patient suitability. However, if the patient does not have ASCVD, the recommendation is to add an additional main drug (sulfonylureas, thiazolidinediones, DPP4 inhibitors, SGLT2 inhibitors, GLP-1 agonists, or basal insulin). Additionally, meglitinides are considered if the patient has allergies or other issues with taking sulfonylureas. As for recently diagnosed patients with elevated Hba1c levels (≥9%), it is recommended to consider starting with dual treatment. Insulin can be considered in those newly diagnosed and with symptoms (hypoglycemia, weight loss, and ketosis), or those with very elevated Hba1c levels (≥10%) and/or glucose levels > 16.7 mmol/L. Other antidiabetic medications, including alpha-glucosidase inhibitors, are used less frequently, primarily due to the high cost and lower efficacy. Choosing a drug should be tailored to the individual, taking into account factors such as its efficacy, adverse effects, benefits, expenses, patient's preference, and overall health (kidney and cardiovascular well-being, Hba1c levels, glucose readings, and weight) [33].

Studies have shown that the efficacy of reducing the HbA1c levels is similar between the different classes of drugs, with a reduction of approximately 1% [34]. Regarding combination therapy (metformin or other primary option with another drug), the reduction in HbA1c is between 0.85 and 1.21%, and adding a third drug decreases levels by 0.53–0.91%; however, having a higher HbA1c at initiation resulted in a larger decrease. Studies also show that the effectiveness of sulfonylureas decreased with time (which may be attributed to the decline of beta cell function in patients over time); and TDZs showed more potency in obese patients as well as in females [34].

### 3.1. The Relevance of Mitochondria in Diabetes

**Figure 2:**
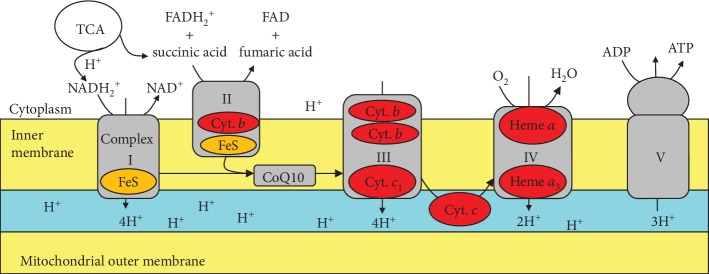
Depiction of the mitochondrial electron transfer system.

Mitochondria are fundamentally linked through the various interconnected pathways to diabetes pathogenesis. The mitochondria generate energy via electron transfer through protein complexes, where the potential difference produced across the membrane is transferred as potential energy to ATP (Figure 2). At the islet *β*-cell level, insulin release is controlled by mitochondrial ATP production. Mitochondrial dysfunction thus decreases ATP and decreases the stimulus-secretion coupling in pancreatic beta cells, which has been suggested in the pathology of type 2 diabetes [35]. Additionally, fuel oxidation in the mitochondria produces reactive oxygen species (ROS), which may contribute to the long-term deterioration of insulin secretion and could be involved in the autoimmune destruction of type 1 diabetes. The generation of ROS may also interfere in insulin signalling in muscle, contributing to insulin resistance in type 2 diabetes; moreover, the mitochondrial function has been shown to affect insulin sensitivity in the muscle, liver, and adipose tissue. Genetic mutations such as mDNA deletions can lead to much rarer cases of “mitochondria diabetes,” which further suggests a fundamental relationship between the two. Interestingly, studies with skeletal muscle biopsy samples taken from patients with type 2 diabetes revealed that mitochondrial complex I activity was reduced by approximately 40%. Furthermore, microarray analysis has displayed that the expression of genes involved in mitochondrial oxidative metabolism is reduced in patients with type 2 diabetes [26, 35].

### 3.2. What Is 5-Aminolaevulinic Acid?

**Figure 3:**
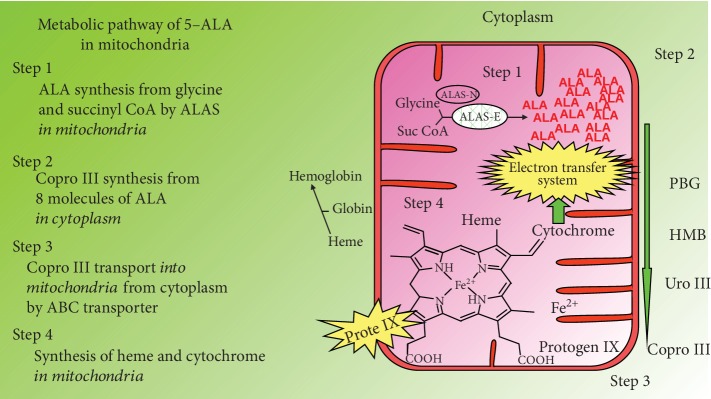
Biosynthesis pathway of heme in mitochondria of animal cell.

5-Aminolaevulinic acid (5-ALA) is a natural delta amino acid present in the human body, which is synthesized from glycine and succinyl CoA by mitochondrial ALA synthase in the mitochondria of animal cells (Figure 3). The polymerization of eight molecules of 5-ALA produces a precursor of heme, protoporphyrin IX, in the mitochondria after going through many intermediate steps in the cytoplasm. Heme is then generated by the insertion of ferrous ion into PpIX in the mitochondria and can carry out electron transfer in the mitochondrial inner membrane, which is coupled with a TCA cycle and produces ATP (Figure 2).

**Figure 4:**
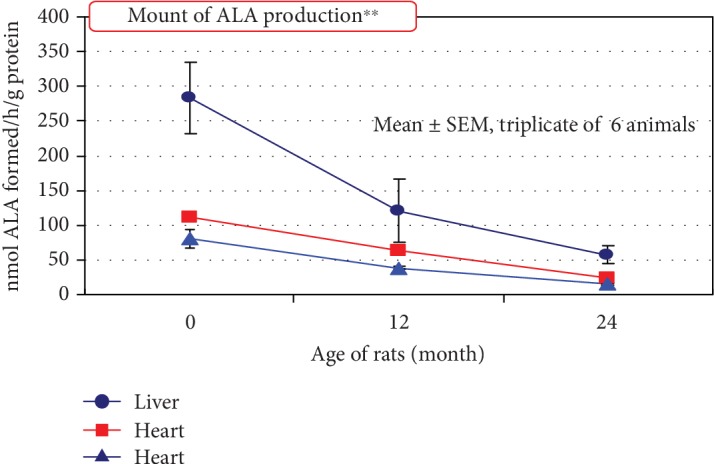
Decrease of 5-ALA synthesis with age: the amount of 5-ALA produced in the human body decreases with age. Aging might occur because the amount of heme decreases [45] and the use of nutrition and oxygen for energy production becomes inefficient [46].

Studies have suggested that 5-ALA enhances aerobic energy metabolism, especially cytochrome *c* oxidase activity as well as protein expression in the mitochondria. 5-ALA also induces heme oxygenase-1 [HO-1] expression, a rate-limiting enzyme in heme metabolism, in the kidney and in cultured cells. It generates cytoprotective products such as bilirubin and carbon monoxide and has been shown to play a role in reducing hyperglycemia in several diabetes models [36]. 5-ALA stores in the human body decrease with age (Figure 4), leading to decreased HO-1 expression within cells. Several studies have proven the safety of normal doses of 5-ALA supplementation in both animals and humans, as well as anti-inflammatory effects [37].

### 3.3. Use of 5-ALA in Type 2 Diabetes

A recent study performed on mice demonstrated that subjects heterozygous for ALA synthase 1 gene (ALAS1) suffer from abnormal glucose levels and insulin resistance, while the skeletal muscle cells showed an increase mitochondrial activity. They further found that 5-ALA deficiency resulted in heme deficiency because of decreased glucose uptake in myocytes of mice with absent ALAS1 or those with a block in heme synthesis, ultimately suggesting that ALA deficiency in vivo can lead to impaired glucose levels and insulin resistance [38].

Studies have shown the effects of 5-ALA on glucose control in both prediabetic and diabetic patients (Table 1). A double-blinded, randomized prospective parallel-group study in Hawaii was conducted on 154 individuals who were prediabetic (HbA1c 5.8-7%) which were randomized equally to three groups: (1) low-dose 5-ALA supplement (15 mg), (2) high-dose 5-ALA (50 mg), and (3) control (placebo capsule of identical size and colour). In those taking 5-ALA (ALA/SFC) supplements for 3 months, 2-hour post-OGTT glucose levels declined significantly compared to those taking the placebo (*p* = 0.02). There was a stronger correlation in those with baseline glucose intolerance—with 2-hour post-OGTT glucose measurements greater than 140 mg/dL (*p* = 0.005 and *p* = 0.02 for the low- and high-dose group, respectively). Similar trends were observed for HbA1c, but results were of borderline significance (*p* = 0.07) [34]. Another study in Japan was conducted with on individuals who demonstrated mild hyperglycemia (fasting plasma glucose 105-125 mg/dL (5.8 mmol/L–6.9 mmol/L) or hemoglobin (Hb)A1c 6.1%-7.1%); they were randomly assigned to 4 groups with different doses of ALA: the results showed reduced fasting and 2 h post-OGTT plasma glucose levels after 3 months [39], and both studies reported no adverse effects [40, 41].

A study on the metabolic effects of 5-ALA and its use in diabetes was performed in Otsuka Long-Evans Tokushima Fatty (OLETF) rats at intragastric doses of 20 and 300 mg kg(-1) d(-1) for 6 weeks. The results showed improved glucose intolerance, hypertriglyceridemia, and hyperleptinemia in OLETF rats at better rates than the administration of an equivalent dose of metformin, in accordance with reductions in food intake and weight. Additionally, retroperitoneal fat weight decreased, and the mitochondrial content of the fat was markedly reduced, essentially showing that 5-ALA reduced the visceral fat mass and mitochondrial content of adipocytes [42].

An additional single-blinded randomized placebo-controlled and parallel-group comparison study in Japan was done to determine the safety of the combined use of 5-ALA–P-SFC (5-aminolevulinic acid (phosphate) sodium ferrous citrate) with oral hypoglycemics, including the potential for hypoglycemia. Forty-five patients were randomly assigned to receive either 15 mg 5-ALA–P-SFC (sodium ferrous citrate), 50 mg 5-ALA–P-SFC (sodium ferrous citrate), or a placebo (*n* = 15). The supplement or placebo was administered for 12 weeks followed by a 4-week washout. The main assessment was the safety of the drug and the occurrence of hypoglycemic attacks, and the secondary endpoint was changed in fasting blood glucose and HbA1c levels.

The results showed no adverse events or abnormalities. Although no significant decrease in fasting blood glucose was detected, there was a decrease in HbA1c at 4 and 8 weeks in the 15 mg 5-ALA group. Significant decreases in HbA1c were not seen in the 50 mg 5-ALA group, although a tendency to decrease after 4 weeks was seen [43].

**Figure 5:**
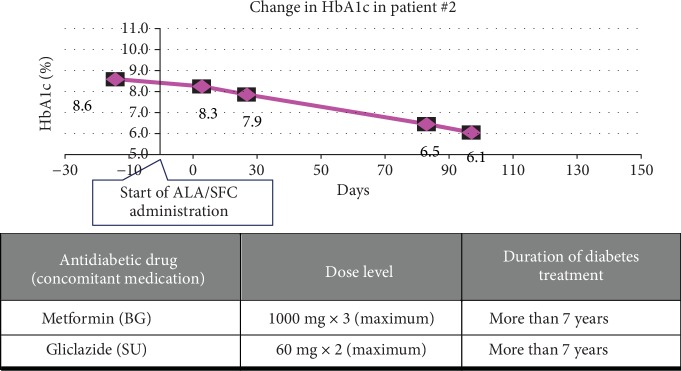
Typical case of the Bahrain study. HbA1c in the patient under long-term therapy with high/maximum doses of oral hypoglycemic agents was dramatically improved by 5-ALA/SFC supplement (HbA1c decreased from 8.6 to 6.1 in 12 weeks) (unpublished data).

A prospective, randomized, single-blind, placebo-controlled, dose-escalating pilot clinical trial assessed the safety of 5-ALA with SFC at doses up to 200 mg 5-ALA/229.42 mg SFC per day. The study enrolled 53 patients living in Bahrain with type 2 diabetes mellitus that was unresponsive to the use of one or more antidiabetic drugs. There was no significant difference in incidence of adverse events reported with the most common events reported being gastrointestinal in nature, consistent with the known safety profile of 5-ALA in patients with diabetes. No significant changes in laboratory values and no difference in hypoglycemia between patients receiving 5-ALA and placebo were noted. Overall, the current results support that usage of 5-ALA with SFC (up to 200 mg per day taken as 2 divided doses) is safe in patients taking concomitant oral antidiabetic medications and may offer benefits in the diabetic population. When used in tandem with other forms of diabetes medications (specifically sulfonylureas), 5-ALA is expected to stimulate insulin sensitivity in patients who are deemed “insulin resistant” (continuous high HbA1c levels) by enhancing glucose metabolism in the TCA cycle (Figure 5). This particularly benefits those who have renal complications and the elderly where HbA1c levels are not lowered despite high dosages of sulfonylureas. Larger studies enrolling a greater number of patients over longer periods of time are required to further define the effect of 5-ALA with SFC on glycemic control in this population [12].

As described above, all the medications currently used in the treatment of diabetes exhibit adverse effects; the benefit of using 5-ALA would be the absence of such extreme adverse effects, as its safety and tolerance have been shown in clinical studies. It would also be comparatively cheaper compared to some of the oral hypoglycemic agents, such as the more costly GLP-1 agonists and others described above; however, further studies are needed to show the efficacy of 5-ALA in the treatment of diabetes, including factors such as reduction of HbA1c levels. Regarding safety, ALA has been avoided in patients with porphyria due to the pathophysiology of the disease; generally, studies with 5-ALA have a consistently low level of adverse events, with some studies reporting no adverse events experienced [40, 41]. Events reported were usually mild, with no serious adverse events reported—these results are summarized in Tables 1 and 2.

## 4. Conclusion

The provided analysis of the different mechanisms of action of antidiabetic medications reveals there are various means through which glycemic control can be achieved in the human body. Each class of drugs has a different mechanism of action, potency of glycemic control, and adverse reactions. Studies support the involvement of mitochondria in the pathology of diabetes, including its dysfunction, and the production of damaging free reactive oxidative species, as well as decreased insulin secretion and increased resistance. Assessing the mechanism of action of 5-ALA, an amino acid produced in the mitochondria and a newer supplement that has recently emerged on the market containing 5-ALA, studies show its effects on the mitochondria and its other metabolic effects including heme oxygenase-1 (HO-1) expression and cytoprotective products. Such findings suggest that the association between diabetes and the mitochondria could be utilized in managing type 2 diabetes, especially given the evidence of mitochondrial dysfunction to increase related risk factors [4]. This is supported by the recent studies showing the safety of the 5-ALA supplement in the diabetic population, especially with the absence of hypoglycemic events so far in the studies conducted. Although currently the role of 5-ALA is as a supplement, it could lend a supporting role toward the successful treatment of diabetes in the future.

## Figures and Tables

**Table 1:** Clinical studies for evaluation of safety and efficacy of 5-ALA/SFC on prediabetes and type 2 diabetes mellitus subjects (OHAs: oral hypoglycemic agents).

Study	Subjects	Reference
(1) Hawaii study	Prediabetes	[40]
(2) Hiroshima study	Prediabetes	[41]
(3) Tokyo study	Type 2 DM treated with OHAs	[43]
(4) Bahrain study	Type 2 DM treated with OHAs	[12]

**Table 2:** A summary of the type 2 diabetes medications, their adverse events, and severe adverse event possibilities.

Medications and adverse events	Observed adverse event (>5% of patients) **(>5% of patients)**	Serious adverse event
5-ALA	(i) Common cold symptoms (ii) Menstrual pain (iii) Diarrhea (iv) Headache [12]	None

Metformin	(i) Diarrhea (ii) Nausea (iii) Abdominal discomfort (iv) Metallic taste (v) Vitamin b12 deficiency	Lactic acidosis in patients lowered eGFR (contraindicated if <30 mL/min per 1.73 m^2^) [21, 47]

DPP4	(i) Dizziness (ii) Headache (iii) Nasopharyngitis (iv) URTI	(i) Severe arthralgia (ii) Acute pancreatitis (iii) Hepatic dysfunction (iv) Serious skin reactions [21, 24]

Sulfonylureas	(i) Weight gain (ii) Dizziness (iii) Headaches (iv) Sulfonamide allergies	(i) Higher mortality after myocardial infarctions (ii) Hypoglycemia [48, 49]

Meglitinides	Weight gain	Hypoglycemia (possibly less than sulfonylureas) [50]

TZDs	(i) Weight gain (ii) Edema (iii) Macular edema (iv) Raised LDL (rosiglitazone) (v) Decrease in hematocrit and hemoglobin when combined with other antihyperglycemic	(i) Bladder cancer (pioglitazone) (ii) Higher rate of myocardial infarctions (rosiglitazone) (iii) Heart failure (iv) Fractures (v) Hypoglycemia [21, 51–53]

Alpha-glucosidase inhibitors	(i) Bloating, flatulence (ii) Diarrhea	(i) Deranged LFTs (acarbose) [21]

SGLT2 inhibitors	(i) Vulvogavinal candidiasis (ii) Urinary tract infections (iii) Hypotension (iv) Dehydration	(i) Fatal urosepsis (ii) Pyelonephritis (iii) Bone fracture (canagliflozin) (iv) Diabetic ketoacidosis [21, 49]

Lipase inhibitors	(i) Abdominal pain (ii) Fecal urgency (iii) Steatorrhea (iv) Vitamin deficiencies (A, D, E, K) (v) Drug interactions (vi) Flu symptoms	(i) Acute kidney injury (ii) Oxalate nephropathy [5, 31, 54]

GLP-1 receptor agonists	(i) Nausea, vomiting (ii) Diarrhea	(i) Pancreatitis (ii) Gallbladder disease (iii) Renal injury [28, 53, 55]
